# *Leptospira* and Bats: Story of an Emerging Friendship

**DOI:** 10.1371/journal.ppat.1005176

**Published:** 2015-11-12

**Authors:** Muriel Dietrich, Kristin Mühldorfer, Pablo Tortosa, Wanda Markotter

**Affiliations:** 1 Department of Microbiology and Plant Pathology, Faculty of Natural and Agricultural Sciences, University of Pretoria, Pretoria, South Africa; 2 Department of Wildlife Diseases, Leibniz Institute for Zoo and Wildlife Research, Berlin, Germany; 3 UMR PIMIT, Université de la Réunion, CNRS 9192, INSERM 1187, IRD 249, Reunion Island, Sainte Clotilde, France; The University of North Carolina at Chapel Hill, UNITED STATES

A growing number of recent studies have highlighted bats as a reservoir for *Leptospira* bacteria, pointing out the potential role of bats in the epidemiology of the most widespread zoonotic disease in the world [1]. Because leptospirosis is a largely neglected disease, a number of unanswered questions remain about the ecology and evolution of *Leptospira*, especially those associated with bats. Here we summarize what has been recently learned about this emerging but enigmatic host–pathogen association. We show how this system can provide exciting new opportunities to obtain insights into the evolutionary ecology of bat-borne pathogens and propose future directions to disentangle the role of bats in human leptospirosis.

## What Do We Know, Briefly, about Leptospirosis and *Leptospira*?

Leptospirosis is a bacterial disease of humans and animals caused by pathogenic spirochetes of the genus *Leptospira*. In humans, leptospirosis is an important (re-)emerging zoonosis of global public health concern [1], although tropical regions display the highest human incidence [2]. Over 500,000 human cases of severe leptospirosis are thought to occur each year worldwide, with a mortality rate of over 10%. Asymptomatic or subclinical human infections are common, making leptospirosis likely far more prevalent than currently diagnosed or recognized [3].

*Leptospira* are a complex of highly diversified bacteria comprising 22 species that include pathogenic (*Leptospira interrogans*, *L*. *kirschneri*, *L*. *borgpetersenii*, *L*. *mayottensis*, *L*. *santarosai*, *L*. *noguchii*, *L*. *weilii*, *L*. *alexanderi*, *L*. *kmetyi*, and *L*. *alstonii*), intermediate (i.e., species of unclear pathogenicity: *L*. *broomii*, *L*. *fainei*, *L*. *inadai*, *L*. *licerasiae*, *L wolffii*), and saprophytic (i.e., free-living and generally considered not to cause disease: *L*. *biflexa*, *L*. *idonii*, *L*. *meyeri*, *L*. *terpstrae*, *L*. *vanthielli*, *L*. *wolbachii*, *L*. *yanagawae*) species [4]. Alongside genetic characterization, serological classification (based on bacterial cell surface antigens) differentiates nearly 300 *Leptospira* serovars, of which more than 200 are considered pathogenic [5]. However, serovars are not indicative of the taxonomic relation among strains because one serovar may belong to more than one species (e.g., *L*. *interrogans* serovar Hardjo and *L*. *borgpetersenii* serovar Hardjo) and multiple serovars occur within the same species [6]. A wide variety of mammals can be infected, but rodents are recognized as significant reservoir hosts. Pathogenic and intermediate leptospires reside in the kidneys of infected animals and are spread through the excretion of urine into the environment [1]. Thus, contaminated soil or water as well as direct contact with infected animals are the main sources of leptospirosis.

## To What Extent Are Bats Infected with *Leptospira*?

**Fig 1:**
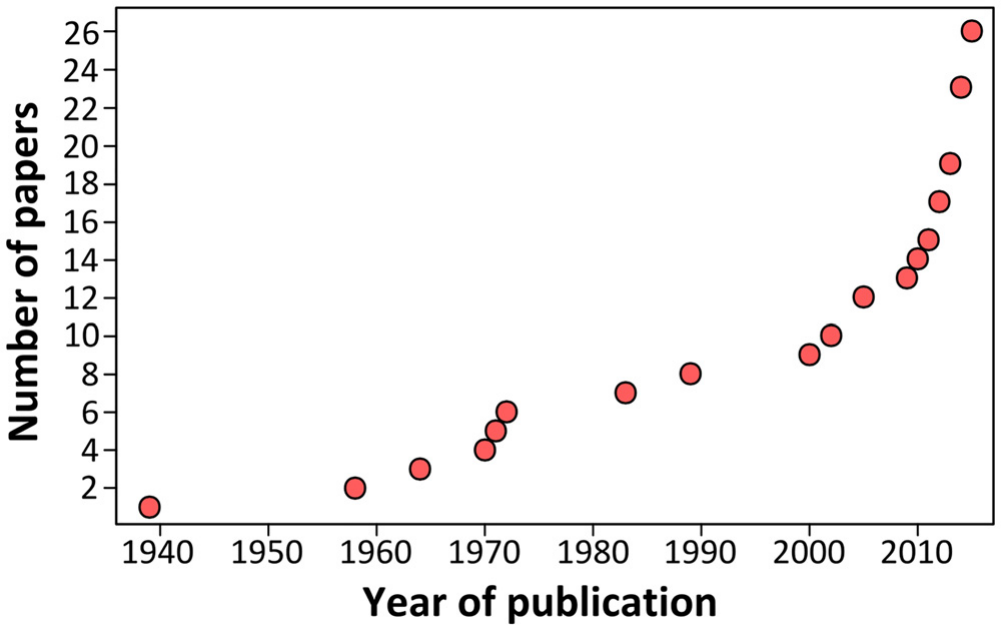
Cumulative number of publications investigating *Leptospira* infection in bats over the past 75 years.

**Fig 2:**
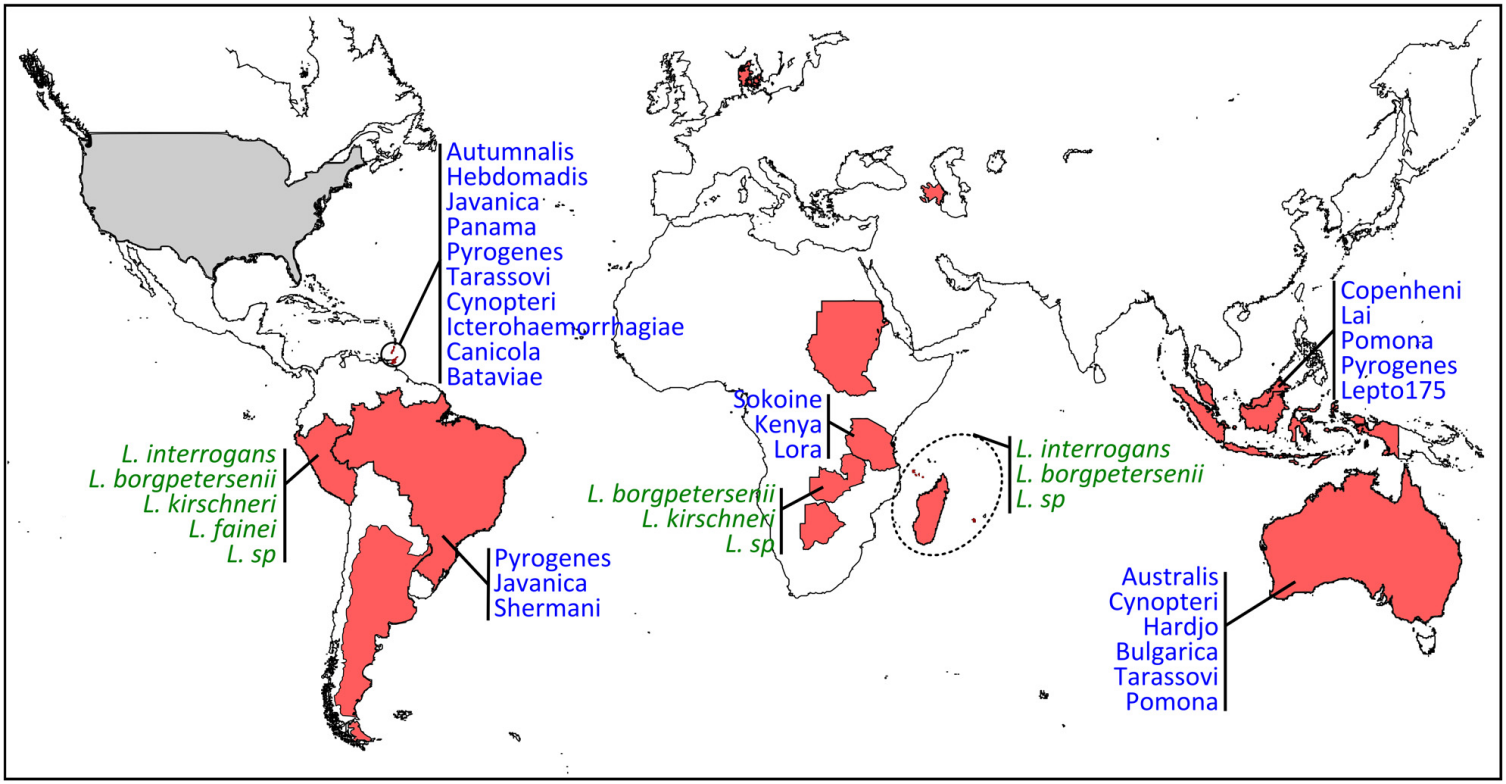
Geographic distribution and diversity of *Leptospira* in bats. Countries are highlighted in red when PCR and/or serology were found positive (corresponding to the following bat families: Phyllostomidae, Mollosidae, Vespertillionidae, Hipposideridae, Miniopteridae, Nycteridae, Mormoopidae, Pteropodidae) and in grey for negative results (Thyropteridae). When available, *Leptospira* diversity is shown in green when genetic data have been used for species identification and in blue when serological analyses have been performed for serovar determination.

Growing scientific interest in bats as reservoirs of pathogens and the global importance of human leptospirosis have led to the emergence of investigations on the presence of *Leptospira* in wild bats during the last few years (Fig 1). Different techniques such as dark-field microscopy, serology by Microscopic Agglutination Test (MAT), PCR detection, and bacterial culture have been used. To date, *Leptospira* infection has been evidenced in over 50 bat species belonging to 8 of the 9 investigated bat families, encompassing various geographical regions in the tropics and subtropics [7–30] as well as Europe, although to a limited extent (Fig 2) [31,32]. *Leptospira* prevalence and seroprevalence in bat populations vary according to bat species and location. Given that bat sampling is often opportunistic, small sample sizes may account for the bias observed in the results. Moreover, a recent study revealed that the prevalence of *Leptospira* excretion in bat urine is highly variable over time, ranging from 6% to 45% within the same colony over a five-month period [20]. Thus, infection dynamics leading to variations in *Leptospira* shedding should be taken into account when bat populations are monitored for prevalence.

## Which *Leptospira* Infect Bats, and When?

There is increasing evidence that bats are infected by highly diverse leptospires, especially in tropical regions with high bat species richness [8,16,19]. Based on genetic identification, bats are infected by at least four species, i.e., *L*. *interrogans*, *L*. *borgpetersenii*, *L*. *kirschneri*, *L*. *fainei*, and likely yet-undescribed genetic clades (Fig 2) [8,19,28]. The use of multilocus sequence analysis has largely improved our view of *Leptospira* diversity in bats and has shown strong host specificity [19] as well as coinfection with multiple *Leptospira* [16,28]. The evolution of bat-borne *Leptospira* diversity and host specificity is probably linked to both cospeciation and host-switching events [33] but also to ecological features such as colony density, feeding behavior, and migration [34]. According to whole-genome [35] and field-based studies [36], which suggest that different *Leptospira* species have evolved towards different modes of transmission, bat species roosting in high-density colonies may be, for example, primarily infected by *Leptospira* dependent on host-to-host transmission, such as hypothesized for *L*. *borgpetersenii* [35].

Dynamics of *Leptospira* infection in bat populations remains largely overlooked. Bat roosting behavior is thought to favor *Leptospira* transmission via urine [20]. Indeed, the reproduction and aggregation behavior of bats within their roosts have been shown to be linked to active *Leptospira* transmission, leading to high rates of infection in maternity colonies [20]. As demonstrated for RNA viruses, increased prevalence during seasonal bat reproduction may thus be associated with higher risk of spillover [37–40]. However, based on current research, there is very little evidence to disentangle whether bat-borne *Leptospira* persist within the host and/or in the environment, as well as whether they are maintained in nature by perpetuation within and between bat colonies [38]. Field monitoring of *Leptospira* excretion in natural bat populations suggests that bats may develop an immune response after acute infection and then stop excreting *Leptospira* [20]. In contrast, observation of natural *Leptospira*-infected bats in Denmark showed that leptospires are able to colonize the renal tubules of bats followed by continuous excretion in urine up to five months. This would indicate that chronic infection may occur in bats [31], as already characterized in chronic asymptomatic animal carriers such as rats [41].

## What Is the Public Health Risk of Bat-Borne *Leptospira*?

Because of their abundance and spatial distribution, bats may contribute to the global maintenance and dissemination of pathogenic leptospires. However, the role of bats as carriers of strains of leptospires associated with human leptospirosis remains uncertain. Direct transmission of bat-borne *Leptospira* to humans has already been suggested, but never evidenced, following a case of serologically confirmed human leptospirosis after bat exposure [42]. Contact with urine and contaminated water is the main form of disease transmission. Human encroachment into bat habitats as well as increasing urbanization, which facilitates bat roosting in artificial structures, are likely to increase the opportunity for bat-borne *Leptospira* spillover [34]. Indeed, evidence of leptospiral infection of kidneys has already been reported in bats roosting in schools and houses [10,16].

Indirect transmission of bat-borne *Leptospira* to humans may also occur through spillover between bat-borne *Leptospira* and other animal hosts, in particular ground-dwelling species such as rodents that reside or forage under bat roosts [8,13,15]. Such transmission between bats and rodents has already been suggested, as *L*. *interrogans*, a typical rodent-borne *Leptospira* species, has been evidenced in insectivorous and frugivorous bats [8,16]. Elucidating the ecological conditions that may favor bat-borne *Leptospira* transmission thus represents a major challenge for public health.

## What Are Future Directions for Research into Bat-Borne *Leptospira*?

The widespread pattern and enigmatic features of *Leptospira* infection in bats represent a challenging opportunity to study the evolutionary ecology of bat-borne infectious agents of possible importance for public health. While other studies mostly focus on viruses, the study of transmission cycles involving bats and bacterial pathogens in particular will provide an original system to understand general patterns of bat-borne pathogen epidemiology.

As a model system, continued research on the ecology of host and bacteria is necessary. It has been recently shown that *Leptospira* excretion in bats can be highly dynamic [20], but ecological factors that drive spatial and temporal variations of infection remain uncertain. For example, what are the roles of environmental factors such as weather seasonal patterns in the transmission dynamics of *Leptospira* in bat populations? Is *Leptospira* infection in bats maintained through epidemic episodes during the bat reproductive season in maternity colonies, or does it persist endemically within any single local population? Do males play a particular role in dispersing *Leptospira* among colonies compared to phylopatric females? Some of these questions can be addressed using long-term data sets by monitoring bat population dynamics, *Leptospira* excretion, and immune response in bat colonies. Noninvasive urine sampling should be preferred, as it allows the collection of a high number of samples while limiting the disturbance of colonies [20]. This will require the validation of urine shedding as a good proxy of renal infection, as recently demonstrated in rats [43]. Parallel investigation of rodent populations in the vicinity of bat colonies would be necessary to assess potential exchanges between these two animal hosts, as already shown for other infectious agents such as paramyxoviruses [44,45].

Improvement of bacterial culture from noninvasive bat samples (such as urine) would be a crucial step for understanding *Leptospira*–bat associations. First of all, it would improve genetic characterization of bat-borne strains and thus provide a more comprehensive picture of *Leptospira* evolution in bats. Secondly, bacterial isolates would allow experimental studies to investigate chronic manifestations in bats, as already demonstrated for rodents [4], as well as the assessment of the survival of bat-borne *Leptospira* in soil and water, in order to determine the role of environment as a source of infection. Animal models would further enable assessment of host specificity and virulence of bat-borne strains and the potential for possible spillovers. Finally, the development of serological diagnostic tests, designed to express a narrow specificity towards bat-borne strains, will allow us to assess the potential exposure of rodent and domestic animal populations to bat-borne *Leptospira* and to determine the burden of acute and asymptomatic *Leptospira* infection in humans from bat origin.
